# Psychosocial burden in nurses working in nursing homes during the Covid-19 pandemic: a cross-sectional study with quantitative and qualitative data

**DOI:** 10.1186/s12913-022-08333-3

**Published:** 2022-07-26

**Authors:** Susanne Schulze, Sibille Merz, Anne Thier, Marie Tallarek, Franziska König, Greta Uhlenbrock, Matthias Nübling, Hans-Joachim Lincke, Michael A. Rapp, Jacob Spallek, Christine Holmberg

**Affiliations:** 1 Faculty of Health Sciences Brandenburg, University of Potsdam, Potsdam, Germany; 2 Institute of Social Medicine and Epidemiology, Brandenburg Medical School Theodor Fontane, Brandenburg an der Havel, Germany; 3 Department of Public Health, Brandenburg University of Technology Cottbus-Senftenberg, Senftenberg, Germany; 4 Freiburg Research Centre for Occupational Sciences (FFAW), Freiburg, Germany; 5 Social and Preventive Medicine, University of Potsdam, Potsdam, Germany; 6 Faculty of Health Sciences Brandenburg, Brandenburg University of Technology Cottbus -Senftenberg, Senfenberg, Germany; 7 Faculty of Health Sciences Brandenburg, Brandenburg Medical School Theodor Fontane, Brandenburg an der Havel, Germany

**Keywords:** COPSOQ, Nurses, Nursing home, Psychosocial burden, Mixed-methods study, Covid-19

## Abstract

**Background:**

The Covid-19 pandemic led to increased work-related strain and psychosocial burden in nurses worldwide, resulting in high prevalences of mental health problems. Nurses in long-term care facilities seem to be especially affected by the pandemic. Nevertheless, there are few findings indicating possible positive changes for health care workers. Therefore, we investigated which psychosocial burdens and potential positive aspects nurses working in long-term care facilities experience during the Covid-19 pandemic.

**Methods:**

We conducted a mixed-methods study among nurses and nursing assistants working in nursing homes in Germany. The survey contained the third German version of the Copenhagen Psychosocial Questionnaire (COPSOQ III). Using Welch’s t-tests, we compared the COPSOQ results of our sample against a pre-pandemic reference group of geriatric nurses from Germany. Additionally, we conducted semi-structured interviews with geriatric nurses with a special focus on psychosocial stress, to reach a deeper understanding of their experiences on work-related changes and burdens during the pandemic. Data were analysed using thematic coding (Braun and Clarke).

**Results:**

Our survey sample (*n* = 177) differed significantly from the pre-pandemic reference group in 14 out of 31 COPSOQ scales. Almost all of these differences indicated negative changes. Our sample scored significantly worse regarding the scales ‘quantitative demands’, ‘hiding emotions’, ‘work-privacy conflicts’, ‘role conflicts’, ‘quality of leadership’, ‘support at work’, ‘recognition’, ‘physical demands’, ‘intention to leave profession’, ‘burnout’, ‘presenteeism’ and ‘inability to relax’. The interviews (*n* = 15) revealed six main themes related to nurses’ psychosocial stress: ‘overall working conditions’, ‘concern for residents’, ‘management of relatives’, ‘inability to provide terminal care‘, ‘tensions between being infected and infecting others’ and ‘technicisation of care’. ‘Enhanced community cohesion’ (interviews), ‘meaning of work’ and ‘quantity of social relations’ (COPSOQ III) were identified as positive effects of the pandemic.

**Conclusions:**

Results clearly illustrate an aggravation of geriatric nurses’ situation and psychosocial burden and only few positive changes due to the Covid-19 pandemic. Pre-existing hardships seem to have further deteriorated and new stressors added to nurses’ strain. The perceived *erosion of care*, due to an overemphasis of the technical in relation to the social and emotional dimensions of care, seems to be especially burdensome to geriatric nurses.

**Supplementary Information:**

The online version contains supplementary material available at 10.1186/s12913-022-08333-3.

## Introduction

Since early 2020, the Covid-19 pandemic has been one of the most important public health topics worldwide. With 510.270.667 confirmed cases of Covid-19 and 6.233.526 Covid-19-related deaths globally up until April 29th 2022 [1], this crisis challenges health systems all over the world.

Due to the high infection rate, the increasing number of hospitalized patients with severe disease progression and the implementation of intensified hygiene measures, health care professionals’ workload and work-related strain increased during the pandemic. Especially nurses, who work in close proximity to and have most contact with Covid-19 patients, suffer from heightened burden [2, 3] and experience stigmatization [4]. Systematic reviews and meta-analyses concerning the mental health of nurses during the Covid-19 pandemic [5, 6] indicate that a substantial proportion of nurses globally suffer from anxiety (pooled prevalences between 32 and 37%), stress (41–43%), depression (32–35%) and sleep disturbances (38–43%). Nurses in long-term care facilities may be especially affected by the virus, due to factors like older age and comorbidities of the residents, location and size of the facility and insufficient or reduced staffing levels [7, 8], which have contributed to the high morbidity and mortality rates in nursing homes due to Covid-19 [9, 10]. Although research focusing on nursing home staff (e.g. compared to hospital staff) is still scarce [11], first results indicate that nurses working in long-term care facilities seem to be particularly prone to emotional strain, poor mental health and overall heightened burden [2, 11–13], with often pre-existing precarious working conditions worsening the situation [12, 14].

Despite these negative impacts on nurses, there are findings that suggest the Covid-19 pandemic had some positive impacts on health care practitioners, like a better recognition or support at the workplace [15]. Beyond this, it may also lead to positive changes in the nursing sector, like a strengthening of nurses’ professional role or removal of barriers for nursing practice [16, 17].

Against this backdrop, we aimed to evaluate which psychosocial burdens and potential positive aspects nurses working in long-term care facilities experience during the Covid-19 pandemic.

## Methods

We conducted a study with a focus on nurses’ work-related burdens during the Covid-19 pandemic using qualitative and quantitative data collection and analysis methods. The quantitative part consisted of a standardized and anonymous paper-pencil questionnaire distributed among nursing staff working with residents in long-term care facilities in the state of Brandenburg, Germany. For the qualitative part, we conducted semi-structured interviews with geriatric nurses^1^ in order to reach an in-depth understanding of work-related changes and burdens during the first phases of the pandemic. The study was approved by the ethics committee of the Brandenburg Medical School Theodor Fontane (MHB).

### Recruitment

Eligible for participation were geriatric nurses and nursing assistants working in long-term care facilities in rural and urban regions of Brandenburg state. To ensure a defined study area, we identified four regions with particular urban or rural characteristics, in which we contacted all nursing home facilities. To identify all nursing homes within the target regions, we conducted a comprehensive search, using an open-access register [18] and an additional database created by the Institute of Social Medicine and Epidemiology (MHB). The management of these facilities were approached by study staff for participation (e-mail and telephone). Overall, 37 out of 58 facilities agreed to take part (rural region 1: 8/13; rural region 2: 9/17; urban region 1: 13/18; urban region 2: 7/10). Between August and October 2020 (i.e. within the interim period between the first two waves and at the beginning of the second wave of the Covid-19 pandemic in Germany) study materials containing study information and the anonymous questionnaire were sent to the participating facilities and distributed to all nurses and nursing assistants working on long-term care wards. Questionnaires were returned individually by mail using enclosed pre-paid envelopes.

When approached for completion of the survey, potential study participants also received a written invitation to participate in a telephonic, semi-structured qualitative interview aiming at exploring in more depth the dynamics and lived realities of working in long-term geriatric care during the pandemic. This recruitment strategy failed to generate study participants leading to a changed sampling through the professional networks of the authors. Sample criteria were broadened such that care home nurses from all regions of Brandenburg were eligible to participate. Interviews were conducted in June 2021 (during the interim period between the third and fourth wave of the pandemic in Germany).

### Data collection

#### Questionnaire

The survey consisted of several parts, the most important for the aim of this study being the German version of the Copenhagen Psychosocial Questionnaire, third edition (COPSOQ III, see next section). In order to guarantee anonymity, few sociodemographic questions (age, gender), as well as questions on the facility of work were added. Moreover, the Perceived Stress Scale 10 [19] and sociodemographic assessments and assessment of effects of the pandemic on perceived stress using a questionnaire developed in the Addiction Research Consortium [20] completed the survey. Due to our research question, this paper solely focusses on the results for COPSOQ III and the semi-structured interviews.

#### COPSOQ III

The Copenhagen Psychosocial Questionnaire (COPSOQ) is an internationally accepted and widely used instrument for the evaluation of various psychosocial factors at work within diverse occupational fields. It combines selected aspects of several important psychosocial theories related to occupational strain, like (amongst others) the Demand-Control-(Support) [21, 22] or the Effort-Reward-Imbalance model [23]. The latest version, COPSOQ III, was recently evaluated in Germany [24]. It consists of 84 items and 31 scales covering risk and resource factors assigned to the domains “demands”, “influence and possibilities for development”, “social relations and leadership” and “additional factors” as well as “effects” of work related strain. For our study, we adopted one of the original items (“intention to leave Job during the past year”) and added an equivalent question related to the onset of the corona crises (“intention to leave job since beginning of the pandemic”). All items and scales range from 0 to 100 scores, with higher values indicating higher expression on the corresponding scale (i.e. higher values on the “emotional demands” scale indicate that participants perceive their work as more emotionally challenging; higher values for “meaning of work” indicate that the work is perceived as rather meaningful and important). In total, reliability of COPSOQ III scales proved to be good or even very good as 28 of its 31 scales show Cronbach’s α ≥ 0.7 and homogeneity is on a satisfying to even good level for 24 scales in terms of inter class correlation as measure of congruence (ICC ≥ 0.5) [24].

#### Interviews

Interviews were conducted by SM and FK using a set of pre-determined, open-ended questions covering the themes listed in Table 1.

**Table 1:** Qualitative data: themes and sample questions

***Themes***	***Sample questions***
Local implementation of political regulations and (additional) measures taken at the care home	Can you describe the measures taken at your facility? How did you feel about them?
Bans on visitors and their impact on staff and residents	Have there been any bans on visitors? If so, how did it affect residents?
Ability to maintain care relationships under the regulations	In your opinion, which consequences did the crisis have on the relationship between nurses and residents?
Tensions between the risk of being infected and infecting others (residents, relatives)	How do you manage the double risk of being at an increased risk of infection and infecting others?
Quality of care during the pandemic	In your opinion, which effects did the crisis have on the quality of care for residents?
Positive impact of the pandemic	Did the pandemic have any positive effects for you? Are there any positive effects in the area of geriatric care?

### Analyses

#### COPSOQ III

Overall, 53 (29.9%) of 177 eligible participants had at least one missing COPSOQ item. At least 1 (0.56%) and no more than 6 (3.4%) values were missing for each separate COPSOQ item. As a result, the proportion of missings individual scale means (due to missing corresponding scale items) varied between 1.1 and 5.7% per scale. In order to further enhance the number of valid cases for our analysis, and in accordance with the usual treatment of missing values within the COPSOQ [24], we hence calculated individual scale means using mean substitution as long as at least 50% of the corresponding items had been answered. Mean substitution did not alter scale means (differences of − 0.32 to 0.68 scale points, see Table S1) nor did it change the results of the final statistical analysis substantially. Supplement 1 (Table S1) provides more information on missing data and a comparison of scale means for the dataset with vs. without mean substitution.

To assess the potential impact of the Covid-19 pandemic on the psychosocial burden of our participants, we compared COPSOQ scale means of our sample with reference values (summary data) of geriatric nurses collected before the pandemic (2015–2019), which were extracted from the German COPSOQ databank and kindly shared by the Freiburg Research Centre for Occupational Sciences (FFAW). The inspection of boxplots revealed few extreme outliers (± 3 IQR) within our sample. However, since all raw data lay within the valid range of values and erroneous data entry was excluded, we kept these scores within the dataset. Additionally, data for almost all COPSOQ scales within our sample were non-normally distributed according to KS-Tests and at least for some scales we found heterogeneity of variances (F-max tests). Therefore, we compared scale means of our sample and the reference group using Welch’s tests, since it is recommended for unequal variances and still performs well when variances are equal [25]. Additionally, we calculated effect sizes (Hedges’ g). All analyses were performed using IBM SPSS Statistics 27 and Microsoft Excel 2016.

#### Interviews

Interviews were conducted by phone or via the online conference tool Webex as chosen by participants. They were recorded on a digital audio device and transcribed verbatim. Interview and transcription logs were kept to record initial observations during the interview and transcription processes respectively. Data was managed using the qualitative software MAXQDA© and thematically analysed [26]. All material was initially coded inductively by SM for key themes ([26] , p. 82) relating to the key research question of how nurses experienced their working conditions during the Covid-19 pandemic. Key themes were reviewed and refined to discern the specifics of each theme in an iterative process, including comparing the COPSOQ results with initial themes to refine them further [26].

Analysis was regularly discussed in the research team.

## Results

### COPSOQ

#### Sample

Two hundred three of 1770 distributed questionnaires were sent back (response rate: 11.5%). There were participants within the sample that did not specifically belong to the nursing staff, primarily care aides,^2^ social workers or managers. Because of the focus of this study, and since a direct comparison of “nursing staff” vs. “other staff” revealed significant differences between group means in seven out of 32 scales of the COPSOQ (see supplement, Table S2), we excluded any participants who did *not* indicate to work as a nurse or nursing assistant (*n* = 24) or did not want to reveal their professional background (*n* = 2). This reduced our sample to *N* = 177 participants (see Table 2 for sample characteristics).

**Table 2:** Sample characteristics

*Age, years (M (SD))*	44.1 (11.7)
*Gender, female (*n (%))	145 (82.9)
*Profession (n (%))*	
nurses	34 (19.2)
geriatric nurses	70 (39.5)
nursing assistants	34 (19.2)
geriatric nursing assistants	39 (22.0)
*Region (n (%))*	
Urban region 1	67 (37.9)
Urban region 2	37 (20.9)
Rural region 1	37 (20.9)
Rural region 2	36 (20.3)
*Operator of facility (n (%))*	
Public	12 (6.8)
Non-profit	87 (49.2)
Private	78 (44.1)

*N* = 177

#### COPSOQ results

COPSOQ mean scores, standard deviations, number of valid cases per scale and results of the Welch’s tests comparison of our sample (GN_Brb) and the reference group of geriatric nurses from the German COPSOQ database (Ref_GN_COPSOQ) are shown in Table 3. For example, in the COPSOQ *effect* section our sample showed higher values for *burnout symptoms*, *intention to leave profession*, *presenteeism* and *inability to relax* in comparison to the reference group. Reasons for this deterioration include among others increased demands for *hiding emotions, work privacy conflicts*, *role conflicts* and *work environment*. The mean score of the additional item *intention to leave (since beginning of the pandemic)* did not differ significantly from the original COPSOQ scale (*t* (175) = 0.834, *p* = .406) (Table 3).

**Table 3:** COPSOQ Scale means, standard deviations, n and Welch’s t-test results for GN_Brb vs. Ref_GN_COPSOQ

	***GN_Brb***			***Ref_GN_COPSOQ***			***Welch’s t-test***			
	*n*	*M*	*SD*	*n*	*M*	*SD*	*t*	*df*	*p*	*g*
**Demands**										
Quantitative Demands	175	59.10	18.76	807	54.86	19.80	2.68	264.87	**.008**	0.22
Emotional Demands	176	74.57	19.03	808	72.51	22.61	1.26	293.12	.209	0.09
Hiding Emotions	176	53.48	24.37	808	48.51	23.17	2.47	248.58	**.014**	0.21
Work Privacy Conflicts	176	51.21	26.50	796	41.73	28.91	4.22	275.02	**<.001**	0.33
Dissolution	176	35.44	22.84	573	36.26	25.55	0.40	320.86	.687	−0.03
**Influence and Possibilities for Development**										
Influence at Work	176	44.15	23.79	807	46.98	22.75	1.44	249.59	.151	−0.12
Degrees of Freedom (Breaks/ Holidays)	176	56.82	25.35	810	58.61	25.59	0.85	258.32	.397	−0.07
Possibilities for Development	176	62.95	18.45	811	65.56	19.80	1.68	269.69	.094	−0.13
Meaning of Work	176	90.98	13.61	813	86.82	18.23	3.44	326.85	**.001**	0.24
Commitment to Workplace	176	63.92	28.41	809	62.58	25.32	0.58	239.17	.563	0.05
**Social Relations and Leadership**										
Predictability of Work	175	56.86	22.29	811	58.65	21.68	0.97	250.05	.334	−0.08
Role Clarity	176	79.05	15.95	810	77.49	17.23	1.16	271.23	.248	0.09
Role Conflicts	176	51.96	23.92	804	41.73	23.67	5.15	255.52	**<.001**	0.43
Quality of Leadership	173	52.19	25.49	798	57.92	23.97	2.71	242.36	**.007**	−0.24
Support at Work	175	68.00	22.54	805	71.98	20.50	2.15	240.56	**.033**	−0.19
Feedback	176	47.37	23.85	805	48.68	22.24	0.67	245.94	.506	−0.06
Quantity of Social Relations	174	62.36	27.11	790	53.83	29.90	3.68	273.77	**<.001**	0.29
Sense of Community	176	74.15	18.01	806	76.33	18.30	1.45	259.93	.147	−0.12
Unfair Treatment	171	23.10	26.01	801	20.97	24.40	0.98	238.11	.328	0.09
Trust and Justice	176	62.78	18.13	806	63.88	18.78	0.72	263.51	.471	−0.06
Recognition	174	47.41	29.94	563	55.11	27.81	3.01	271.62	**.003**	−0.27
**Additional Factors**										
Work Environment/ Physical Demands	176	52.24	19.22	609	43.84	20.73	5.02	302.71	**<.001**	0.41
Job Insecurity	176	16.03	21.37	806	18.42	21.63	1.34	259.23	.180	−0.11
Insecurity over Working Conditions	175	30.76	27.14	570	27.53	23.88	1.42	262.04	.158	0.13
**Effects**										
Intention to leave Profession/ Job (past 12 Months)	176	25.85	28.55	797	18.50	22.86	3.20	227.02	**.002**	0.31
Intention to leave Profession/ Job (since Covid-19 pandemic)^a^	176	24.64	31.35	–	–	–	–	–	–	
Job Satisfaction	176	61.47	17.34	807	64.27	16.62	1.95	249.90	.052	−0.17
Work Engagement	173	67.58	21.10	571	70.84	18.89	1.82	261.01	.070	−0.17
General Health	171	64.68	20.76	764	67.88	20.11	1.83	246.49	.068	−0.16
Burnout Symptoms	173	61.01	19.80	810	52.12	20.85	5.30	259.94	**<.001**	0.43
Presenteeism	173	51.30	30.89	808	45.48	26.83	2.30	230.79	**.022**	0.21
Inability to Relax	173	51.45	29.98	569	45.52	27.83	2.31	268.30	**.021**	0.21

*GN_Brb* Geriatric nursing staff in long-term care facilities during the corona crisis, Brandenburg, *Ref_GN_COPSOQ* Reference sample of geriatric nurses from the German COPSOQ database, 2015–2019

^a^additional self-inserted scale; *g* Hedges’ g

### Interviews

#### Sample

Overall, 15 care nurses who worked in nursing homes from two rural regions in Brandenburg participated in the interviews. Of those 14 had experienced outbreaks in their respective work environments at the time of the interview. Of those, four occupied managerial positions and one was an ancillary nurse. Twelve interviewees were female and three were male, with an age range of 20 to 60. All interviewees were educated until at least their GSCE; all but one had either completed vocational training or had received a higher degree (equivalent to BSc in Nursing).

#### Key themes

We identified seven key themes relating to the Covid-19 pandemic: overall working conditions; concern for isolated residents; management of relatives; inability to provide terminal care and perform mourning rituals; tensions between being infected and infecting others; the technicisation of care through strict adherence to hygiene protocols; and enhanced community cohesion. Experiences of burden and distress are well described in the following statement made by an ancillary nurse during the interviews as ‘the worst thing I have seen in 21 years working in geriatric care’ (CP11).

### Overall working conditions

Interviewees spoke about both the physical and the broader psychosocial implications of working under the hygiene protocols adopted in the nursing home. Staff shortages led to longer working hours, the need to maintain distance to colleagues meant long queues at the changing room or the prohibition of taking joint breaks. In particular, interviewees reported of added pressure due to the mandatory wearing of personal protective equipment (PPE, i.e. masks, visors, hazmat suits, gloves, caps and overshoes). Beyond the mere physical implications, changing into and out of PPE often several times a day significantly impeded everyday care routines, particularly during active outbreaks where spatial segregation of infected and non-infected residents was impossible. In contrast, time with residents and performing actual care work such as preventing social and emotional deprivation through social interaction and conversation was limited, making interviewees feel as if they let residents ‘fall through the cracks’ (CP01):

Well, preventing residents’ social isolation was impossible. You didn’t even have the time to really engage with residents, because due to the fact that we had to change [into PPE] completely anew for each resident, we lost a lot of time (CP06)*.*

Interviewees working in managerial positions also reported that adhering to, implementing and monitoring regulations that sometimes changed day-by-day increased pressure on them and fundamentally changed their daily routines.

### Concern for residents

Most interviewees expressed deep concern for residents being isolated for long periods. Due to restrictions on group activities, shared meals and visitors, residents were largely confined to their rooms, especially during active Covid-19 outbreaks on their ward. Interviewees often witnessed grave deteriorations in residents’ physiological and mental health during this time, and told us of their concern for their well-being. Having to isolate residents contrasted their understanding of the social conditions of health and well-being, and contradicted the significance of direct, often sensory, interaction with a range of interlocutors (staff, relatives, other residents, nursing aides). Many interviewees worried about this not only from a nursing science standpoint, but also from a deeply human, emotional perspective:

Well in sum, one could also observe, residents who were full of life before, who had visitors at least twice a week, they completely collapsed, they almost died on us. They withdrew, they deteriorated cognitively, and often very much so. How I feel about this? Well, pretty negative (CP01)*.*

This was especially difficult when interacting with cognitively compromised residents who often did not understand the necessity of the regulations, and were even more vulnerable to the effects of isolation.

### Management of relatives

Interviewees reported increased stress due to the ban on relatives visiting residents, in place across most are homes in the beginning (March–May 2020) and during the third wave (December 2020–March 2021) of the pandemic. While visitors usually support and unburden care staff through time spent with individual residents, this support suddenly ceased to exist. Worse, relatives often became an additional burden for interviewees. Relatives needed to be kept informed about the condition of residents, and interviewees provided emotional support for relatives when the resident’s status deteriorated. Some found it especially stressful to inform relatives about residents’ immediate death. Moreover, some relatives were unaccepting of the regulations and blamed interviewees for barring them from visiting. In many instances, interviewees reported of insults and abuse from relatives, which they had to mediate or fend off. After restrictions on visits had eased and when palliative visits were granted, geriatric nurses were responsible for ensuring that residents as well as visitors adhered to the regulations. In many cases, interviewees had to test visitors for Covid-19 at the care home, adding to their workload:

In that moment, where relatives aren’t coming anymore, we are the main contact person for the residents, for their worries, their problems. We are then also responsible for helping shape their leisure time. Those are things that we didn’t really do earlier, because relatives would support us with this (CP14)*.*

However, some interviewees reported feeling more appreciated by relatives and that the shared suffering during the pandemic had strengthened the bond between residents, relatives and staff.

### Inability to provide terminal care

Interviewees found it difficult to provide adequate terminal care for residents. Many reported that they found it extremely difficult to know that residents had to die in isolation, and that they had missed the opportunity to say goodbye to long-term residents with whom they had built strong personal relationships. This tarnished their self-understanding of the kind of palliative care they deemed necessary. Moreover, working conditions, as well as financial pressure to re-fill beds, made it impossible for interviewees to adequately mourn deceased residents. While they admitted that death and dying are natural elements of working in long-term geriatric care, both the quality and quantity of death(s) under Covid-19 were difficult to process. Though tensions had eased at the time of the interview, many realized only then that they had not fully processed residents’ death. Some had been offered supervision or counselling by their supervisors, but most preferred casual conversations with colleagues to make sense of the events.

The residents who were infected with Covid and then also died from it, we could not do them justice anymore. Terminal care or not leaving them alone in those moments, we could not do justice to this. They often laid alone in their rooms. Some older residents still had relatives visiting, for the acute, terminal phase. But many had no one, and this really did something to us (CP07)*.*

### Tensions between being infected and infecting others

While some interviewees were anxious about contracting the virus or had already recovered from Covid-19 at the time of the interview, some were more concerned about infecting relatives or residents at the care home. This was especially worrisome for those who cared for vulnerable family members at home; where possible, interviewees maintained physical distance to those family members. For most, the risk of infection and transmission and the guilt attached to it was a constant companion during their work. Even after infections had eased and most residents and staff members were vaccinated, some interviewees told of the recurring anxiety every time they were tested, and the stigma attached to being ‘the one who brought the virus into the care home’.

Sometimes it really does put you under a lot of pressure. To always be exposed to this situation, to think ‘oh hopefully, I don’t have anything. Oh God, oh God, I hope I’m not the one introducing it [to the care home]. That really does something to you (CP12)*.*

### The technicisation of care

All interviewees reported that the regulations had a negative impact on their ability to provide adequate emotional or psychosocial care, though some noted that the provision of basic care such as personal hygiene was successfully maintained. Conversations, group activities and interpersonal interaction all fell prey to the increased time spent on adhering to the regulations, changing into and out of PPE, testing visitors and staff and interacting with public health institutions. Adherence to hygiene protocols often meant that residents could no longer exercise autonomy, for example about when to get up in the morning or when to take meals. In direct interaction with residents, masks, PPE and restrictions on physical contact significantly limited meaningful interaction. Many interviewees thus experienced an overdetermination of the technical/practical dimensions over the emotional dimensions of care, described as an act of de-individualisation and de-humanisation:

This not-being-able-to-be-there was extremely burdensome to me … This, just to know that they’re sitting in their rooms alone. Over months, they didn’t have anyone, we’re talking about months here. And every day the same, out of bed, get washed, eat at the table, clear the table. This was so unloving, so inhumane! (CP07)

These tensions were amplified when caring for residents with decreased cognitive capacities who did not comprehend the necessity of the regulations and seldom adhered to them, frequently leaving their rooms to mingle with others. Moreover, cognitively impaired patients are even more dependent on stable relationships, mimics and physical touch, all hampered by staff shortages, the use of temporary staff and the mandatory wearing of full PPE. Interviewees observed how these patients suffered from the lack of personal interaction and intimacy, either silently through weight loss or by becoming physically and verbally aggressive but could do relatively little to help, causing great stress for both resident and staff. Some interviewees admitted that they sometimes transgressed the regulations by taking their masks off to allow more ‘human’ interaction, or by taking extra time with residents in need as the situation became unbearable.

### Enhanced community cohesion

When queried about positive effects of the pandemic, however, respondents particularly emphasized enhanced solidarity and community cohesion among the team. While early in the pandemic, some noticed an increasing level of suspicion amongst workers, others reported increased cohesion and a do-it-yourself-mentality due to the lack of governmental support. As soon as infections hit, all respondents observed an elevated team spirit and increased mutual support, including enhanced solidarity with those suffering from long-term effects of a Covid-19 infection. Respondents in managerial positions were worried about attrition or absenteeism but observed the opposite as staff members showed high levels of commitment to residents and colleagues.

A really positive development and very touching was that we, as an institution and also as a team, became a lot closer due to this challenge, and showed a lot more understanding for each other. A new kind of appreciation has developed, this is very, very, positive (CP13)*.*

## Discussion

In 2019, more than 4.1 million people in Germany were in need of long-term care [27]. Most of them received domiciliary care, either by relatives (56%) or by professional care services (24%), and 20% lived in full-time residential care homes [27]. Nevertheless, with about 800.000 employees (65%), the majority of nursing staff within the long-term care sector works in residential care homes [28]. Precarious working conditions, especially of nurses working in the long-term care sector, like insufficient payment, unfavorable employment situation, work-life balance and high workload have been criticized for a long time [29, 30], making elderly care nurses a particularly vulnerable group within the health care system. Especially during the early phases of the pandemic, German nursing homes and their staff have been under immense pressure due to frequent and serious Covid-19 outbreaks in many facilities and the fact that care home residents constituted a substantial proportion of all Covid-19 related deaths [9], adding to nurses’ burden.

Hence, in this study we investigated how changes in care practices, such as the hygiene protocols adopted during the Covid-19 pandemic in Germany, the isolation of residents or the introduction of additional tasks, impacted the working experiences of study participants.

Concerning the COPSOQ, our sample of nursing staff working in long-term care facilities in Brandenburg scored significantly different in 14 out of 31 scales compared to the pre-corona reference sample of geriatric nurses from the German COPSOQ databank. Almost all of these differences reflect negative changes. Effect sizes were small to moderate according to Hedges’ *g*, although 11 scale means exceeded (and the remaining 3 were close to) the threshold of ±5 scores difference, which is seen as a meaningful cut-off for group differences [31]. Results clearly illustrate an aggravation of study participants’ psychosocial burden during the Covid-19 pandemic. This is reflected by significant increases of all negatively connoted scales within the COPSOQ *effect* section: Compared to the reference group, our sample showed higher values for *burnout symptoms*, *intention to leave profession*, *presenteeism* and *inability to relax*. Reasons for this deterioration seem to be diverse and not limited to actual emotional or psychosocial strains. Rather structural factors, like deteriorated working conditions, seem to exacerbate the situation additionally. It is important to note that the pandemic in Germany evolved geographically from the South to the North. So that the first wave in spring 2020 was experienced more severly in Southern regions of the country. More Northern and Northeastern regions, such as Brandenburg, only experienced more severe outbreaks starting in fall of 2020 after the survey had been administered. The interview portion of the study was conducted after the second and third wave had hit the region.

Thus, the qualitative part of this study provided a deeper insight into geriatric nurses’ changes in working routine and experiences during the pandemic. Results revealed that the additional tasks and measures implemented to combat the virus and its spread, like the mandatory wearing of PPE or increased hygiene standards, did not only affect overall workload and working conditions of the interviewees. More importantly, interviewees perceived some kind of *erosion of care* as crucial social and emotional parts of their job were increasingly sidelined. The fact that nurses were barely able to interact socially with residents in a meaningful way, give emotional support or foster residents’ autonomy due to a lack of time and tightened regulations led to high emotional and psychosocial stress. Related to this, the observation of residents’ suffering, growing isolation and resulting deterioration of their physical and mental capacities as well as the fear of infection/transmission were additional stressors for interviewees.

Summarizing the results of our quantitative and qualitative analyses, geriatric nurses in this study expressed overall heightened strain during the Covid-19 pandemic, even before the pandemic hit Brandenburg more severely. While quantitative results of our sample, as compared to the reference group, indicate higher *quantitative demands* (e.g. longer working hours), worse *physical demands* (like physically strenuous work or poor air quality) and more *work-privacy conflicts*, for instance due to energy and time consumed by work which interferes with private life, qualitative results add descriptions of *how* changes in daily routine due to the pandemic had led to additional tasks, like testing or the management of relatives, less time for residents and longer working hours due to staff shortages and intensified hygiene standards. Studies from other countries confirm such an increase of nurses’ workload during the pandemic [32–34] and indicate that higher workloads and other unfavorable working conditions increase psychosocial strain [35], associated to mental health issues like burnout [36].

Indeed, these deteriorated working conditions, specifically the lack of time and the intense hygiene measures, are likely to have fostered actual psychosocial and emotional strains. Most interviewees describe how, due to the changes in care routines and time pressure, the overemphasis of the technical dimensions of care over the crucial social and emotional aspects cause significant stress. Having no time to listen and talk to isolated residents, to meaningfully interact with them and to provide appropriate terminal care contradicts the notion of good care most nurses have internalized. Although the prioritization of the technical aspects of care in situations of reduced time and staffing capacities may be necessary and reasonable to some extent [37], the striking reduction of the social and emotional dimensions of care led to psychological strain and internal role conflicts of nurses [37, 38], which seems to have become more prevalent during the pandemic. Indeed, our quantitative data support this impression, as our sample perceived such *role conflicts* to a significantly higher extent than the reference group. With an increase of more than 10 points, this is one of the most notable quantitative results.

Aside from these emotional and ethical conflicts, interviewees described the observation of residents’ suffering and deterioration due to isolation and limited autonomy as an independent source of stress during the pandemic. Furthermore, the handling of worried or noncompliant relatives, the constant fear to be the one who transmits the virus either to the nursing home or to family and friends and related concerns for stigmatization put additional pressure on them. A recent meta-analysis and systematic review confirms that health care workers indeed experience concerning levels of stigmatization in their direct and broader social environment and that this leads to heightened risks for depression and anxiety [4]. Emotionally demanding situations like these may require nurses to suppress their feelings in order to keep working. In line with this assumption, our sample showed significantly enhanced demands to *hide emotions* compared to the pre-pandemic reference group. This is concerning, since such negative mechanisms of emotion regulation have previously been found to impair psychological well-being and health in people engaging in emotional labor [39].

Our study sample showed significantly increased mean scores in all negatively connoted scales of the COPSOQ domain *effects* compared to the reference group, expressing higher levels of *burnout*, *presenteeism*, *inability to relax* and *intention to leave profession*. Simultaneously, for the rather positive connoted scales (*job satisfaction, work engagement* and *general health*) the comparison revealed decreased scores within our sample, though none of these differences reached significance. Within our study, *burnout* was the effects scale with the highest increase compared to the reference sample. Again, qualitative data support this finding as nurses frequently described examples of emotional as well as physical exhaustion. This is supported by Galanis et al. [36], who found that, amongst other factors, increased workload, longer working hours, working in a high-risk environment and decreased social support are associated with higher burnout rates in nurses during the pandemic. Since our participants seem to perceive a lack of support especially by superiors, as they rated the scales of *recognition* (by the management), *quality of leadership* and *support at work* significantly lower compared to the reference group, all of these risk factors apply to our sample. Therefore, an increase of burnout symptoms is reasonable.

Intuitively, *intention to leave profession* (within past year) is, with 25 of 100 possible points, valued relatively low within our sample. However, it outranges the corresponding mean score of the reference group as well as a large German sample from diverse occupational settings [24], and is at the upper edge of the spectrum in the German nursing sector [40–42]. Results of the NEXT study showed that, amongst others, higher quantitative demands and more work-privacy conflicts (as observed within our sample) increase intention to leave the profession in nurses [29, 43]. This may point to an actual increased intention to leave within our sample due to the changed working conditions during the pandemic although the mean score of the additional item *intention to leave (since beginning of the pandemic)* did not differ significantly from the original COPSOQ scale.

*Presenteeism* was operationalized by asking participants how often they come to work despite feeling unwell or sick [24]. The mean of this scale was also enhanced within our group compared to the reference sample. Indeed, within the qualitative interviews, managers stated that a suspected raise in sick leave did not occur. This is noteworthy given the high infection risk of nurses [44] and the multitude of distressing experiences described above, but might be explained by the intensified team spirit interviewees emphasized.

The incapacity to stop thinking about work during time off was measured with the single-item scale *inability to relax* and was significantly more common in our sample compared to the reference group. This is a probable observation, since many aspects of the Covid-19 pandemic infiltrate work as well as private life, such as the constant fear of transmitting the virus from private social contacts to residents or vice versa, as described not only by our interviewees but also by healthcare professionals in other studies [45–47].

Despite the obvious negative impacts of the Covid-19 pandemic on nurses’ working conditions and psychosocial wellbeing, our results revealed some *positive aspects*, too. First, interviewees frequently emphasized *the enhanced social cohesion* within the nursing teams, and other studies [48] as well as several aspects within our COPSOQ data underline this notion. For instance, our sample rated the *quantity of social contacts* significantly higher than the pre-pandemic reference group, which may indicate a high perceived support by colleagues. Second*, meaning of work* was significantly higher in our sample during the pandemic than in the pre-pandemic reference sample. This is remarkable, as this was the highest rated positive scale within the reference sample, leaving very limited scope to exceed. Perhaps the perceived importance of work was further enhanced by the publics’ attention, recognition of nurses’ merit and the resulting gratitude towards nursing staff. Especially during times of isolation and quarantine, nurses were often residents’ most important social contacts, which was also described by our interviewees. This experience and the gratitude of residents and relatives might have further increased feelings of professional identity and responsibility [49], thereby enhancing meaning of work. A recent scoping review identified enhanced team relationships and finding meaning in work as important resources to handle ethical challenges during a pandemic [48]. Both might be sources of resilience, helping nurses deal with the multitude of hardships experienced during the Covid-19 pandemic [6, 40, 50].

### Strengths & Limitations

Our study provides insight into the situation of nurses working in nursing homes in Germany, a population that is still rarely studied despite their supposed disposition for heightened work-related strain during the Covid-19 pandemic. The COPSOQ provided insight into a wider range of work-related stressors and their effects on psychosocial burdens. Furthermore, the application of a frequently used, validated instrument like the COPSOQ facilitates comparison with results from other populations or countries. Moreover, we conducted semi-structured interviews with a specific focus on geriatric nurses’ concrete experiences, which allowed for a deeper understanding of the psychosocial strains directly related to the pandemic.

Nevertheless, there are some limitations to consider. First, we did not have pre-pandemic COPSOQ data of our sample, hindering a direct within-subjects comparison and evaluation of the genuine effect the pandemic had on work-related psychosocial strain within our sample. We solved the problem the best possible way by contrasting our results against a large German reference group with identical occupational backgrounds evaluated in the years before the pandemic. Nevertheless, we cannot fully exclude possible pre-existing differences between the two samples. However, most of the identified differences between our sample and the reference group were supported by results gained within the qualitative arm of this study and external findings, strengthening the assumption that the found discrepancies are genuine effects due to the pandemic. Second, despite wide distribution of the questionnaire, we reached a comparatively small sample size, which might have led to selection bias. Especially highly burdened nurses may not have participated, which in turn could have resulted in an underestimation of the impact the crisis had on the target group. Considering the high number of stressors we identified, this assumption makes the need for support and de-escalation in care even more urgent. Third, the COPSOQ was administered at a time when study participants may not yet have had personal outbreak experiences at work. Due to data protection we cannot link the survey data to the nursing homes. For this reason, we do not know in what ways the experience of actual outbreaks would influence the study results. Fourth, we were not able to recruit a subsample of interviewees from our original quantitative sample. Nevertheless, our interviewees were nurses working in nursing homes within the state of Brandenburg, comparable in age and interviewed during the Covid-19 pandemic. Thus, they most likely had similar work-related experiences, although most of the interviewees, except for three, experienced outbreaks at work.

## Conclusion

The increased amount of bureaucratic documentation, the wearing of PPE, the lack of time for residents or the impossibility to provide terminal care address the deeply human dimensions of care work that relies on relationality, humanness and vulnerability. The inability to provide good care in this sense might be the biggest psychosocial burden geriatric nurses have endured during the Covid-19 pandemic. Moreover, considering the unbalanced proportion of psychosocial stressors and resources identified in this study, the often pre-existing disadvantageous working conditions within the nursing sector and the negative impacts these hardships already have on nurses’ mental health worldwide [6, 50, 51], the compelling need for support and relief for this profession becomes even more urgent. Many of the strains found here (e.g. high workload, job demands, stress etc.) have previously been associated with high turnover intention in nurses all over the world [52]. In order to maintain high quality care and a healthy workforce, policy makers and institutions should take measures to reduce nurses’ strains by offering psychosocial support, enhancing working conditions and creating conditions that allow nurses to provide the care work they deem respectful, human and necessary.

## Supplementary Information

**Additional file 1.**

## Data Availability

The dataset used and analysed during the current study is available from CH (last author) on reasonable request.

## Abbreviations

COPSOQ: Copenhagen Psychosocial Questionnaire; within this article, this means the third German Version of the COPSOQ
PPE: Personal protective equipment

## Footnotes

1 For the sake of readability of this paper, we use the term “geriatric nurses” for all nurses and nursing assistants working in nursing homes within our sample.

2 Care aides support residents in various fields of their everyday life and foster social activities, rather than carrying out nursing tasks.
