# Supplementary material for: Psychosocial burden in nurses working in nursing homes during the Covid-19 pandemic: a cross-sectional study with quantitative and qualitative data
Source: BMC Health Serv Res. 2022 Jul 26;22:949. doi: 10.1186/s12913-022-08333-3 (PMC9315075; doi:10.1186/s12913-022-08333-3)
Supplement: Supplementary file 1 — Additional file 1. [file 12913_2022_8333_MOESM1_ESM.docx]

**Supplementary material**

**S1 - Missingness: COPSOQ scale means with vs. without single imputation of missings by case mean substitution**

In order to further enhance sample size, we substituted missing items by the mean of the remaining scale items of the same case as long as at least 50% of these scale items were given. In the following table, scale means, SDs, number of valid cases and proportion of missing cases of our sample (n =177) are displayed for the dataset without vs. with case mean substitution.

**Table S1**

|  | **COPSOQ GN_Brb**  **without mean substitution** | | | | | **COPSOQ GN_Brb**  **With mean substitution** | | | | |
| --- | --- | --- | --- | --- | --- | --- | --- | --- | --- | --- |
|  | M | SD | Whole sample (n) | Valid cases n (%) | Missings  n (%) | M | SD | Whole sample (n) | Valid cases n (%) | Missings  n (%) |
| **Demands**  Quantitative Demands | 58.98 | 18.67 | 177 | 167 (94.4) | 10 (5.7) | 59.10 | 18.76 | 177 | 175 (98.9) | 2 (1.1) |
| Emotional Demands | 74.57 | 19.09 | 177 | 175 (98.9) | 2 (1.1) | 74.57 | 19.03 | 177 | 176 (99.4) | 1 (0.6) |
| Hiding Emotions | 53.50 | 24.44 | 177 | 175 (98.9) | 2 (1.1) | 53.48 | 24.37 | 177 | 176 (99.4) | 1 (0.6) |
| Work Privacy Conflicts | 51.21 | 26.57 | 177 | 175 (98.9) | 2 (1.1) | 51.21 | 26.50 | 177 | 176 (99.4) | 1 (0.6) |
| Dissolution | 35.19 | 22.80 | 177 | 173 (97.7) | 4 (2.3) | 35.44 | 22.84 | 177 | 176 (99.4) | 1 (0.6) |
| **Influence and Possibilities for Development**  Influence at Work | 44.12 | 23.91 | 177 | 173 (97.7) | 4 (2.3) | 44.15 | 23.79 | 177 | 176 (99.4) | 1 (0.6) |
| Degrees of Freedom (Breaks/ Holidays) | 57.50 | 24.53 | 177 | 170 (96.1) | 7 (4.0) | 56.82 | 25.35 | 177 | 176 (99.4) | 1 (0.6) |
| Possibilities for Development | 63.10 | 18.41 | 177 | 175 (98.9) | 2 (1.1) | 62.95 | 18.45 | 177 | 176 (99.4) | 1 (0.6) |
| Meaning of Work | 90.93 | 13.63 | 177 | 175 (98.9) | 2 (1.1) | 90.98 | 13.61 | 177 | 176 (99.4) | 1 (0.6) |
| Commitment to Workplace | 63.79 | 28.54 | 177 | 174 (98.3) | 3 (1.7) | 63.92 | 28.41 | 177 | 176 (99.4) | 1 (0.6) |
| **Social Relations and Leadership**  Predictability of Work | 56.79 | 22.37 | 177 | 173 (97.7) | 4 (2.3) | 56.86 | 22.29 | 177 | 175 (98.9) | 2 (1.1) |
| Role Clarity | 78.90 | 15.99 | 177 | 173 (97.7) | 4 (2.3) | 79.05 | 15.95 | 177 | 176 (99.4) | 1 (0.6) |
| Role Conflicts | 52.23 | 23.77 | 177 | 172 (97.2) | 5 (2.8) | 51.96 | 23.92 | 177 | 176 (99.4) | 1 (0.6) |
| Quality of Leadership | 52.60 | 25.19 | 177 | 168 (94.9) | 9 (5.1) | 52.19 | 25.49 | 177 | 173 (97.7) | 4 (2.3) |
| Support at Work | 68.17 | 22.32 | 177 | 172 (97.2) | 5 (2.8) | 68.00 | 22.54 | 177 | 175 (98.9) | 2 (1.1) |
| Feedback | 47.59 | 23.92 | 177 | 171 (96.6) | 6 (3.4) | 47.37 | 23.85 | 177 | 176 (99.4) | 1 (0.6) |
| Quantity of Social Relations | 62.36 | 27.11 | 177 | 174 (98.3) | 3 (1.7) | 62.36 | 27.11 | 177 | 174 (98.3) | 3 (1.7) |
| Sense of Community | 74.42 | 17.97 | 177 | 173 (97.7) | 4 (2.3) | 74.15 | 18.01 | 177 | 176 (99.4) | 1 (0.6) |
| Unfair Treatment | 23.10 | 26.01 | 177 | 171 (96.6) | 6 (3.4) | 23.10 | 26.01 | 177 | 171 (96.6) | 6 (3.4) |
| Trust and Justice | 62.86 | 18.26 | 177 | 173 (97.7) | 4 (2.3) | 62.78 | 18.13 | 177 | 176 (99.4) | 1 (0.6) |
| Recognition | 47.41 | 29.94 | 177 | 174 (98.3) | 3 (1.7) | 47.41 | 29.94 | 177 | 174 (98.3) | 3 (1.7) |
| **Additional Factors**  Work Environment/ Physical Demands | 52.19 | 19.31 | 177 | 173 (97.7) | 4 (2.3) | 52.24 | 19.22 | 177 | 176 (99.4) | 1 (0.6) |
| Job Insecurity | 15.71 | 21.28 | 177 | 174 (98.3) | 3 (1.7) | 16.03 | 21.37 | 177 | 176 (99.4) | 1 (0.6) |
| Insecurity over Working Conditions | 30.94 | 27.12 | 177 | 174 (98.3) | 3 (1.7) | 30.76 | 27.14 | 177 | 175 (98.9) | 2 (1.1) |
| **Effects**  Intention to leave Profession/ Job  (past 12 Months) | 25.86 | 28.72 | 177 | 174 (98.3) | 3 (1.7) | 25.85 | 28.55 | 177 | 176 (99.4) | 1 (0.6) |
| Intention to leave Profession/ Job  (since Covid-19 pandemic)** | 24.93 | 31.42 | 177 | 174 (98.3) | 3 (1.7) | 24.64 | 31.35 | 177 | 176 (99.4) | 1 (0.6) |
| Job Satisfaction | 61.47 | 17.54 | 177 | 170 (96.1) | 7 (4.0) | 61.47 | 17.34 | 177 | 176 (99.4) | 1 (0.6) |
| Work Engagement | 67.54 | 21.16 | 177 | 172 (97.2) | 5 (2.8) | 67.58 | 21.10 | 177 | 173 (97.7) | 4 (2.3) |
| General Health | 64.68 | 20.76 | 177 | 171 (96.6) | 6 (3.4) | 64.68 | 20.76 | 177 | 171 (96.6) | 6 (3.4) |
| Burnout Symptoms | 61.35 | 19.55 | 177 | 171 (96.6) | 6 (3.4) | 61.01 | 19.80 | 177 | 173 (97.7) | 4 (2.3) |
| Presenteeism | 51.30 | 30.89 | 177 | 173 (97.7) | 4 (2.3) | 51.30 | 30.89 | 177 | 173 (97.7) | 4 (2.3) |
| Inability to Relax | 51.45 | 29.98 | 177 | 173 (97.7) | 4 (2.3) | 51.45 | 29.98 | 177 | 173 (97.7) | 4 (2.3) |

Note: GN_Brb: geriatric nursing staff in long-term care facilities during the corona crisis, Brandenburg

**S2 - “Nursing staff” vs. “other staff” – COPSOQ III scales comparison**

We compared the COPSOQ mean scale scores of participants directly involved in the care of residents (i.e. nurses, geriatric nurses, nursing assistants and geriatric nursing assistants; n=177) against the scores of other related professions working with the residents (e.g. care aides, social workers, n=26) using Welch’s t-tests.

**Table S2**

COPSOQ Scale means, standard deviations, n and Welch's t-test results for nursing staff vs. other staff

|  | **Nursing staff** | | | **Other staff** | | | **Welch’s t-test** | | |
| --- | --- | --- | --- | --- | --- | --- | --- | --- | --- |
|  | n | M | SD | n | M | SD | t | df | p |
| **Demands**  Quantitative Demands | 175 | 59.10 | 18.76 | 22 | 42.50 | 18.04 | 4.05 | 27.03 | **<.001** |
| Emotional Demands | 176 | 74.57 | 19.03 | 22 | 73.30 | 21.92 | 0.26 | 25.12 | .796 |
| Hiding Emotions | 176 | 53.48 | 24.37 | 22 | 44.89 | 23.04 | 1.64 | 27.23 | .113 |
| Work Privacy Conflicts | 176 | 51.21 | 26.50 | 22 | 32.95 | 27.22 | 2.97 | 26.23 | **.006** |
| Dissolution | 176 | 35.44 | 22.84 | 22 | 39.77 | 29.79 | -0.66 | 24.18 | .517 |
| **Influence and Possibilities for Development**  Influence at Work | 176 | 44.15 | 23.79 | 22 | 54.55 | 23.95 | -1.92 | 26.45 | .066 |
| Degrees of Freedom (Breaks/ Holidays) | 176 | 56.82 | 25.35 | 22 | 53.41 | 25.35 | 0.60 | 26.53 | .557 |
| Possibilities for Development | 176 | 62.95 | 18.45 | 23 | 70.65 | 15.05 | -2.24 | 31.35 | **.032** |
| Meaning of Work | 176 | 90.98 | 13.61 | 23 | 83.70 | 16.62 | 2.02 | 26.00 | .054 |
| Commitment to Workplace | 176 | 63.92 | 28.41 | 23 | 63.04 | 29.31 | 0.14 | 27.68 | .893 |
| **Social Relations and Leadership**  Predictability of Work | 175 | 56.86 | 22.29 | 23 | 52.17 | 21.54 | 0.98 | 28.56 | .337 |
| Role Clarity | 176 | 79.05 | 15.95 | 23 | 73.91 | 17.82 | 1.32 | 26.81 | .200 |
| Role Conflicts | 176 | 51.96 | 23.92 | 23 | 43.12 | 25.33 | 1.59 | 25.38 | .127 |
| Quality of Leadership | 173 | 52.19 | 25.49 | 23 | 49.28 | 34.05 | 0.40 | 25.38 | .695 |
| Support at Work | 175 | 68.00 | 22.54 | 23 | 58.97 | 25.69 | 1.61 | 26.64 | .120 |
| Feedback | 176 | 47.37 | 23.85 | 23 | 44.02 | 20.94 | 0.71 | 29.99 | .483 |
| Quantity of Social Relations | 174 | 62.36 | 27.11 | 23 | 60.87 | 22.39 | 0.29 | 31.20 | .773 |
| Sense of Community | 176 | 74.15 | 18.01 | 23 | 65.22 | 21.31 | 1.92 | 26.27 | .065 |
| Unfair Treatment | 171 | 23.10 | 26.01 | 23 | 35.87 | 29.02 | -2.01 | 26.97 | .055 |
| Trust and Justice | 176 | 62.78 | 18.13 | 23 | 53.80 | 21.55 | 1.91 | 26.23 | .067 |
| Recognition | 174 | 47.41 | 29.94 | 23 | 43.48 | 28.42 | 0.62 | 28.85 | .540 |
| **Additional Factors**  Work Environment/ Physical Demands | 176 | 52.24 | 19.22 | 23 | 31.16 | 16.80 | 5.56 | 30.05 | **<.001** |
| Job Insecurity | 176 | 16.03 | 21.37 | 23 | 23.55 | 26.43 | -1.31 | 25.90 | .202 |
| Insecurity over Working Conditions | 175 | 30.76 | 27.14 | 23 | 27.17 | 27.20 | 0.60 | 28.07 | .557 |
| **Effects**  Intention to leave Profession/ Job  (past 12 Months) | 176 | 25.85 | 28.55 | 23 | 18.48 | 23.80 | 1.36 | 30.92 | .183 |
| Intention to leave Profession/ Job  (since Covid-19 pandemic)** | 176 | 24.64 | 31.35 | 23 | 12.50 | 21.32 | 2.41 | 35.83 | **.021** |
| Job Satisfaction | 176 | 61.47 | 17.34 | 23 | 64.44 | 21.28 | -0.64 | 25.96 | .527 |
| Work Engagement | 173 | 67.58 | 21.10 | 24 | 76.04 | 17.43 | -2.17 | 33.12 | **.037** |
| General Health | 171 | 64.68 | 20.76 | 23 | 65.65 | 21.71 | -0.20 | 27.69 | .841 |
| Burnout Symptoms | 173 | 61.01 | 19.80 | 24 | 54.86 | 20.55 | 1.38 | 29.25 | .178 |
| Presenteeism | 173 | 51.30 | 30.89 | 24 | 35.42 | 31.20 | 2.34 | 29.60 | **.026** |
| Inability to Relax | 173 | 51.45 | 29.98 | 24 | 47.92 | 31.20 | 0.52 | 29.21 | .606 |

Note: ** additional self-inserted scale
